# Downregulation of death receptor 4 is tightly associated with positive response of EGFR mutant lung cancer to EGFR-targeted therapy and improved prognosis

**DOI:** 10.7150/thno.54824

**Published:** 2021-02-15

**Authors:** Shuo Zhang, Zhen Chen, Puyu Shi, Songqing Fan, Yong He, Qiming Wang, Yixiang Li, Suresh S. Ramalingam, Taofeek K. Owonikoko, Shi-Yong Sun

**Affiliations:** 1 Department of Hematology and Medical Oncology, Emory University School of Medicine and Winship Cancer Institute, Atlanta, Georgia, USA.; 2 Department of Pathology, The Second Xiangya Hospital, Central South University, Changsha, Hunan, China.; 3 Department of Respiratory Disease, Daping Hospital, Chongqing, China.; 4 Department of Internal Medicine, The Affiliated Cancer Hospital of Zhengzhou University, Henan Cancer Hospital, Zhengzhou, Henan, China.

**Keywords:** EGFR inhibitors, osimertinib, death receptor 4, apoptosis, acquired resistance

## Abstract

Death receptor 4 (DR4), a cell surface receptor, mediates apoptosis or induces inflammatory cytokine secretion upon binding to its ligand depending on cell contexts. Its prognostic impact in lung cancer and connection between EGFR-targeted therapy and DR4 modulation has not been reported and thus was the focus of this study.

**Methods:** Intracellular protein alterations were measured by Western blotting. Cell surface protein was detected with antibody staining and flow cytometry. mRNA expression was monitored with qRT-PCR. Gene transactivation was analyzed with promoter reporter assay. Drug dynamic effects *in vivo* were evaluated using xenografts. Gene modulations were achieved with gene overexpression and knockdown. Proteins in human archived tissues were stained with immunohistochemistry.

**Results:** EGFR inhibitors (e.g., osimertinib) decreased DR4 levels only in EGFR mutant NSCLC cells and tumors, being tightly associated with induction of apoptosis. This modulation was lost once cells became resistant to these inhibitors. Increased levels of DR4 were detected in cell lines with acquired osimertinib resistance and in NSCLC tissues relapsed from EGFR-targeted therapy. DR4 knockdown induced apoptosis and augmented apoptosis when combined with osimertinib in both sensitive and resistant cell lines, whereas enforced DR4 expression significantly attenuated osimertinib-induced apoptosis. Mechanistically, osimertinib induced MARCH8-mediated DR4 proteasomal degradation and suppressed MEK/ERK/AP-1-dependent DR4 transcription, resulting in DR4 downregulation. Moreover, we found that DR4 positive expression in human lung adenocarcinoma was significantly associated with poor patient survival.

**Conclusions:** Collectively, we suggest that DR4 downregulation is coupled to therapeutic efficacy of EGFR-targeted therapy and predicts improved prognosis, revealing a previously undiscovered connection between EGFR-targeted therapy and DR4 modulation.

## Introduction

Lung cancer causes one-fifth of all cancer deaths worldwide and is by far the leading cause of cancer death among both men and women 1, 2. The majority of lung cancer patients (80%) suffer from non-small cell lung cancer (NSCLC) with a 5-year survival rate of approximately 19% after diagnosis. Therefore, great efforts have been made to combat lung cancer worldwide over the past decades. The discovery of epidermal growth factor receptor (EGFR) activating mutations represented a paradigm shift in the treatment of NSCLC. Targeting EGFR activating mutations, 90% of which present as an exon 19 deletion (Del19) or exon 21 point mutation (L858R), with first and second generation EGFR tyrosine kinase inhibitors (EGFR-TKIs; e.g., erlotinib, gefitinib and afatinib) and T790M resistant mutation with third generation EGFR-TKIs (e.g., osimertinib; TAGRISSO^TM^ or AZD9291) has provided significant clinical benefit in patients with NSCLC harboring these mutations, representing a successful example for targeted therapy against lung cancer 3, 4. Clinical studies have shown that osimertinib as first-line treatment for untreated advanced NSCLCs with EGFR activating mutations achieved remarkable positive outcomes with superior efficacies over the standard EGFR-targeted therapy 5, 6 and showed clear benefit in overall survival as recently reported 7. Osimertinib is now also an FDA-approved drug for the first-line treatment of advanced NSCLCs with EGFR activating mutations in addition to its application as a second-line treatment of EGFR mutant (EGFRm) NSCLC patients relapsed from first generation EGFR-TKIs due to T790M mutation. Unfortunately, resistance to osimertinib occurs in the clinic, resulting in disease progression and limiting its long-term efficacy 8, 9. Hence, understanding the underlying mechanisms and developing effective strategies to overcome osimertinib resistance is highly desirable and urgently needed in the clinic.

Death receptor 4 (DR4), also known as tumor necrosis factor-related apoptosis-inducing ligand (TRAIL) receptor 1 (TRAIL-R1) or tumor necrosis factor receptor superfamily member 10A (TNFRSF10A), is a cell surface receptor for TRAIL. It is generally recognized that its activation, upon binding to TRAIL, induces apoptosis. Similar to its sibling, death receptor 5 (DR5), TRAIL/DR4 ligation-induced apoptosis involves the specific interaction of trimerized DR4 with the adaptor protein Fas-associated death domain (FADD) via the death domain. The subsequent recruitment of caspase-8 through the death effector domain of FADD leads to caspase-8 activation and ultimately, apoptosis 10, 11. Since cytotoxic T lymphocytes (CTLs) and natural killer (NK) cells can generate and secrete TRAIL, the induction of apoptosis by ligation of endogenous TRAIL with its receptors on cancer cells has been recognized as a critical mechanism accounting for immune surveillance against malignant cells 12-14.

In general, DR4 shares a redundant function with DR5 in mediating TRAIL-induced apoptosis 10, 11. Thus, many agents, including some anticancer drugs, sensitize cancer cells to TRAIL-induced apoptosis through increasing the expression of DR4 and/or DR5 15, 16. However, DR4 does display distinct functions from DR5, such as in mediating apoptosis induced by certain stimuli 17, 18 and in the regulation of cancer cell invasion and metastasis 19, 20, although the underlying mechanisms are largely unknown. Like DR5, DR4 is also a p53 target gene and its expression can thus be regulated in a p53-dependent manner 21, 22. Moreover, several p53-independent mechanisms that positively regulate DR4 expression including AP-1 23, NF-κB 24-26, c-Myc 27 and retinoic acid receptor 28-mediated gene transcription have been suggested by us and others. Some agents increase DR4 expression through these mechanisms. We have recently demonstrated that DR4 expression is positively regulated by MEK/ERK signaling through AP-1-mediated activation of gene transcription and is suppressed upon MEK inhibition 29. In addition to transcriptional regulation, one study suggested that the E3 ubiquitin ligase, membrane-associated RING-CH-8 (MARCH8), interacts with and ubiquitinates DR4, negatively modulating DR4 protein levels including cell surface levels 30.

A previous study has shown that genetic suppression of Fas, a death receptor of the TNF receptor superfamily, sensitizes EGFRm NSCLC cells to erlotinib, primarily due to the inhibition of NF-κB signaling 31. Two recent studies have suggested that TRAIL death receptors including DR4 mediate the production of inflammatory cytokines such as CCL2/MCP1, IL-8 and CXCL1 induced by TRAIL in TRAIL-resistant cancer cell lines through facilitating the formation of a “FADDosome” complex primarily consisting of caspase-8, FADD and RIPK1 and subsequent NF-κB signaling activation 32, 33. This finding is consistent with a previous report that TRAIL death receptors activate NF-κB-dependent cytokine secretion 34. As a consequence, the secreted cytokines will inactivate immune cells, promoting a tumor-supportive immune microenvironment and encouraging tumor growth 32.

The connection between osimertinib or EGFR-targeted cancer therapy and DR4 suppression has not been reported. Given the critical role of MEK/ERK/AP-1 signaling in the positive regulation of DR4 expression as we recently demonstrated 29 and that osimertinib potently suppresses MEK/ERK signaling in EGFRm NSCLC cells 35, we speculated a potential effect of osimertinib on decreasing DR4 expression in EGFRm NSCLC cells. Indeed, we found that osimertinib and other EGFR-TKIs selectively downregulated the levels of DR4 including cell surface DR4 in EGFRm cancer cell lines *in vitro* and *in vivo*. Hence, our findings for the first time connect DR4 downregulation with the efficacy of osimertinib-based or EGFR-targeted cancer therapy, which is in contrast to the known function of DR4 as a pro-apoptotic protein. Furthermore, we also studied the underlying mechanisms, possible biological significance of DR4 suppression by osimertinib and prognostic role of DR4 expression in NSCLC.

## Results

### Osimertinib and other EGFR-TKIs selectively decrease DR4 levels in sensitive EGFRm NSCLC cells

**Figure 1:**
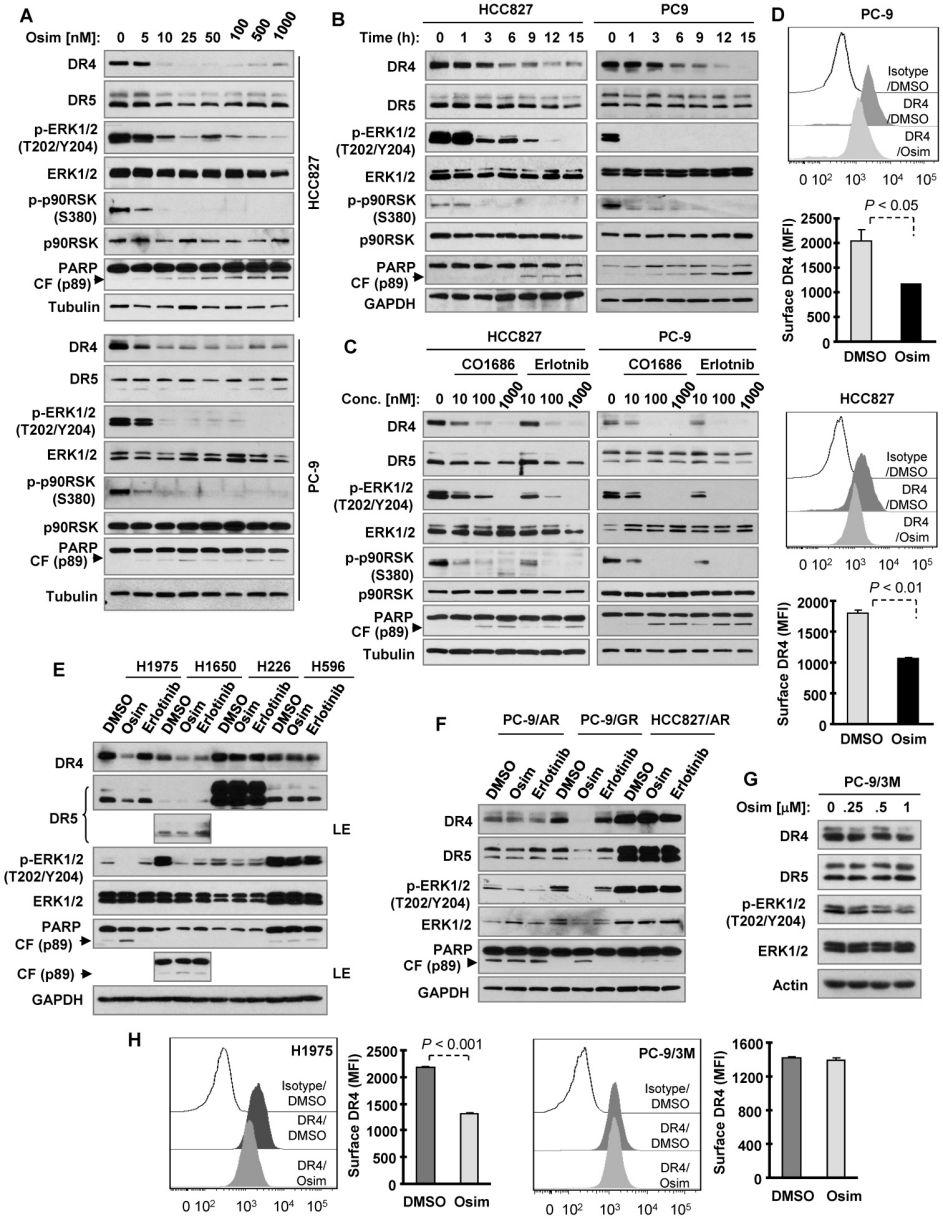
** Osimertinib and other EGFR-TKIs decrease DR4 levels in EGFRm NSCLC cell lines accompanied with induction of apoptosis.** *A-D,* Both PC-9 and HCC827 cells were exposed to the indicated concentrations of EGFR-TKIs for 8 h (*A* and *C*) or to 100 nM osimertinib (Osim) for different times (*B*) or 12 h (*D*). *E*-*G*, The indicated cell lines were exposed to 100 nM of a given EGFR-TKI (*E* and *F*) or to different concentrations of osimertinib for 8 h (*G*). *H*, The tested cell lines were exposed to DMSO or 100 nM osimertinib for 12 h. Total cellular DR4 and cell surface DR4 were detected with Western blotting (*A-C and E-G*) and flow cytometry (*D and H*), respectively. The data in *D* and *H* are means ± SDs of duplicate determinations. CF, cleaved form.

To test our hypothesis that osimertinib may decrease DR4 expression in EGFRm NSCLC cells due to its potent effect on suppressing the MEK/ERK signaling, we first determined the effect of osimertinib on DR4 expression in the two sensitive EGFRm NSCLC cell lines, PC-9 and HCC827. Osimertinib treatment potently and rapidly decreased the levels of DR4 in both cell lines, with little to no reduction of DR5 levels (Figures 1A and 1B). DR4 reduction was achieved by treatment with 10 nM osimertinib (Figure 1A) and occurred early at 3 h of osimertinib treatment (Figure 1B). Importantly, DR4 reduction was accompanied with suppression of phosphorylation of ERK and p90RSK (a known ERK substrate), which occurred even earlier than DR4 reduction (Figure 1B), and PARP cleavage (Figures 1A and 1B), a hallmark of apoptosis. This suggests a tight association between suppression of MEK/ERK signaling and DR4 reduction and between DR4 reduction and apoptosis or a possible causal relationship between MEK/ERK signaling suppression and DR4 downregulation/apoptosis. Besides osimertinib, CO1686 (10-1000 nM) and erlotinib (10-1000 nM) similarly suppressed ERK and p90RSK phosphorylation, decreased DR4 levels, and induced PARP cleavage in these two sensitive EGFRm NSCLC cell lines (Figure 1C). Since DR4 functions as a cell surface protein, we also detected the alteration of cell surface DR4 in cells exposed to osimertinib and found that cell surface DR4 levels were also significantly reduced in both PC-9 and HCC827 cells treated with osimertinib compared with their corresponding DMSO-treated control cells (Figure 1D).

We noted that both osimertinib and erlotinib did not suppress ERK phosphorylation, induce PARP cleavage or decrease DR4 levels in wild-type (WT) EGFR NSCLC cell lines (H226 and H596; Figure 1E), EGFRm NSCLC cell lines with acquired resistance to osimertinib (PC-9/AR and HCC827/AR; Figure 1F) or the EGFRm PC-9 cell line engineered with T790M and C797S mutations that confer resistance to osimertinib (PC-9/3M; Figure 1G). Both agents decreased DR4 levels in the additional H1650 EGFRm NSCLC cell line. However, osimertinib, but not erlotinib, suppressed ERK phosphorylation accompanied with DR4 reduction and PARP cleavage in another H1975 EGFRm NSCLC cell line (containing T790M) and in PC-9/GR, a laboratory-derived gefitinib-resistant EGFRm NSCLC cell line due to T790M mutation (Figures 1E and F). In agreement, osimertinib significantly decreased cell surface DR4 levels in H1975 cells, but not in PC-9/3M cells (Figure 1H). These results together indicate that EGFR-TKIs, particularly osimertinib, suppress MEK/ERK signaling, decrease DR4 expression and induce apoptosis primarily in the sensitive EGFRm NSCLC cell lines.

### Osimertinib quickly and efficiently decreases DR4 levels accompanied with induction of apoptosis and tumor growth *in vivo*

**Figure 2:**
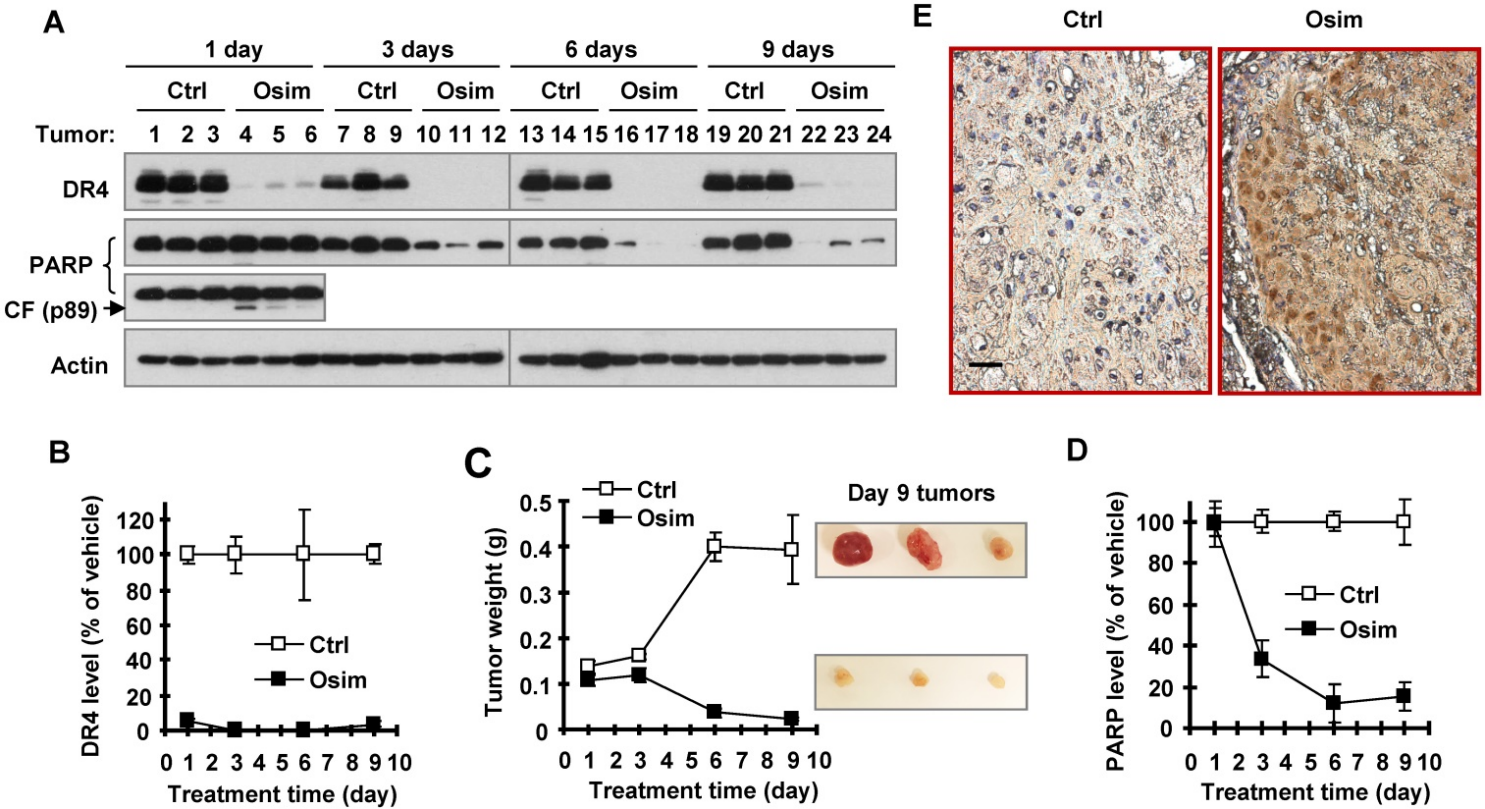
** Osimertinib decreases DR4 levels in an EGFRm NSCLC xenografts *in vivo* accompanied with induction of apoptosis.** PC-9 xenografts at ~ 300 mm^3^ were treated with vehicle control (Ctrl) or osimertinib (Osim; 10 mg/kg; og; once/daily) and collected at the indicated times (N = 3). The given proteins were detected with Western blotting (*A*). The results or band intensities were quantified with NIH Image J software (*B* and *D*) in comparison with tumor weight alteration (*C*). The data presented in *B*-*D* are means ± SEs (N = 3). Control and tumor tissues on day 9 were also stained for cPARP with IHC (*E*). The scale bar is 50 μm. CF, cleaved form.

We next determined whether osimertinib-induced DR4 reduction occurs *in vivo* and is associated with tumor growth. To this end, we detected DR4 modulation in PC-9 xenografts exposed to osimertinib for different times. When tumors grew to approximately 300 mm^3^, vehicle control and osimertinib were administered to mice for 9 consecutive days. Three mice in each group were sacrificed to collect tumors after 1 day, 3 days, 6 days and 9 days of treatment. As observed *in vitro*, osimertinib potently decreased DR4 levels in all tested tumors treated with osimertinib through 1 to 9 days (Figures 2A and 2B). Significant tumor suppression was generated after 6 days treatment (Figure 2C). After 6 and 9 days of osimertinib treatment, tumors were significantly smaller than the initial control tumors (see tumor weights on day 1; Figure 2C), indicating that osimertinib treatment causes tumor regression. Hence, it is clear that DR4 reduction occurred ahead of tumor suppression. Detection of PARP showed a significant reduction in PARP levels in osimertinib-treated tumors after 3 days of treatment, although no clear increases in the levels of cleaved PARP were detected in these tumor samples (Figures 2A and 2D). In tumor tissues exposed to 1-day osimertinib treatment, amounts of the pro-form of PARP were not significantly decreased; however increased levels of cleaved PARP were clearly detected (Figure 2A). Using immunohistochemistry (IHC) with antibody specifically against cleaved PARP (cPARP), we detected increased cells positive for cPARP staining in tumor samples treated with osimertinib for 9 days, but not in the control tissue (Figure 2E). These results collectively demonstrate that osimertinib induces apoptosis in xenografted tumors, which likely occurs after DR4 reduction, but before tumor shrinkage.

### Osimertinib suppresses AP-1-dependent DR4 transcription

**Figure 3:**
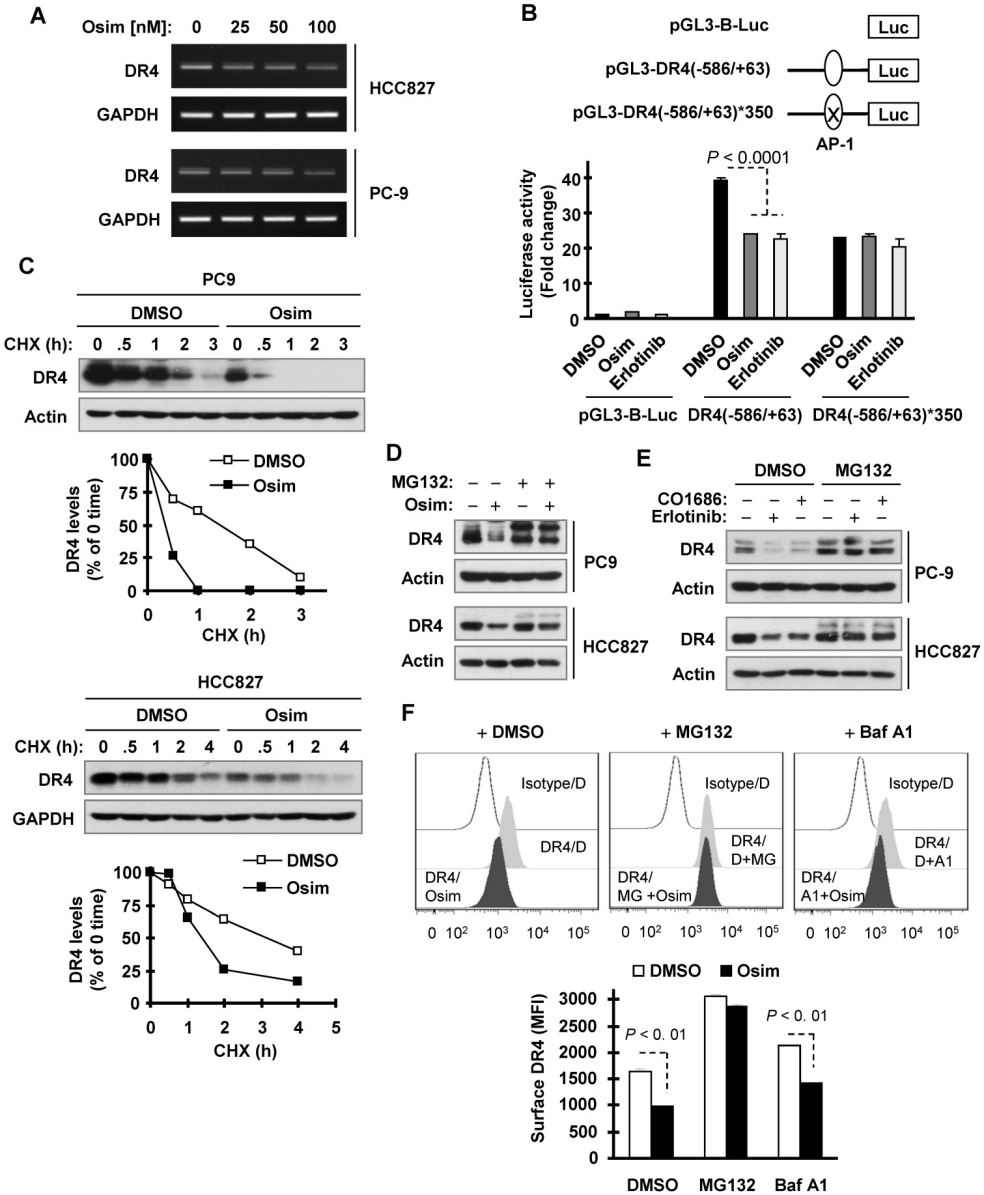
** Osimertinib decreases DR4 mRNA levels, inhibits AP-1-dependent DR4 transactivation, and facilitates proteasomal degradation of DR4.** *A*, PC-9 and HCC827 cells were exposed to the indicated concentrations of osimertinib (Osim) for 6 h. DR4 mRNA levels were detected by RT-PCR. *B,* PC9 cells were transfected with the given DR4 reporter plasmids. Approximately 18 h after transfection, the cells were exposed to 100 nM osimertinib or erlotinib for another 10 h and lysed for luciferase activity assay. The data are mean ± SDs of triplicate determinations. *C*, PC-9 and HCC827 cells were exposed to DMSO or 100 nM osimertinib for 8 h, followed by the addition of 10 µg/ml CHX. Cells were then harvested at the indicated times post CHX for Western blotting. DR4 levels were plotted relative to those at time 0 of CHX treatment after being quantified by NIH Image J software and normalized to Actin or GAPDH. *D* and *F,* The indicated cells were pre-treated with 10 µM MG132, or 50 nM Baf A1 for 1 h and then co-treated with 100 nM osimertinib, CO1686 or erlotinib for an additional 6 h. Total cellular and cell surface DR4 were detected with Western blotting (*D* and *E*) and flow cytometry (*F*), respectively. D, DMSO; MG, MG132; A1, Baf A1.

We then wanted to define the mechanisms by which osimertinib downregulates DR4 expression. We recently showed that DR4 expression is positively regulated by MEK/ERK signaling through AP-1-mediated activation of gene transcription and is suppressed upon MEK inhibition 29. Since osimertinib effectively inhibits MEK/ERK signaling, we then asked whether osimertinib downregulates DR4 expression through this mechanism. We first determined the effect of osimertinib on DR4 mRNA modulation and found that osimertinib indeed decreased DR4 mRNA levels (Figure 3A), suggesting a transcriptional level of modulation. Moreover, both osimertinib and erlotinib significantly suppressed the activity of a DR4 promoter with an active AP-1 binding site, but not one with a mutated or inactivated AP-1 binding site (Figure 3B), indicating that these EGFR-TKIs inhibit AP-1-dependent DR4 transactivation. We also observed that osimertinib decreased the levels of p-c-Jun and c-Jun in both PC-9 and HCC827 cells in time- and concentration-dependent manners (Figure S1). RNA sequencing (RNA-seq) analysis confirmed the suppressed expression of *DR4* (Figures S2A and S2B), *c-JUN*, *FOSL* (*FRA-1*) and *FOS* genes, which encode critical component proteins of AP-1 (Figure S2A and S2C). These results collectively suggest that osimertinib-induced DR4 reduction likely involves the suppression of AP-1-mediated gene transcription.

### Osimertinib induces DR4 proteasomal degradation

Because DR4 is regulated at a posttranslational level through degradation 30, 36, we further determined whether osimertinib modulates DR4 stability through affecting its degradation. Using the cycloheximide (CHX) chase assay, we found that DR4 was degraded much faster in osimertinib-treated PC-9 cells than in DMSO-treated PC-9 cells. The same result was also generated in HCC827 cells (Figure 3C). Thus, it is clear that osimertinib facilitates DR4 degradation. The presence of the proteasome inhibitor, MG132, rescued the DR4 reduction caused by osimertinib, CO1686 or erlotinib in both PC-9 and HCC827 cells (Figures 3D and 3E). However, the presence of the lysosome inhibitor, chloroquine (CQ) or bafilomycin A1 (Baf A1) only partially prevented DR4 reduction induced by osimertinib although both agents potently elevated basal levels of DR4 (Figure S3). Consistently, MG132, but not Baf A1, substantially increased basal surface levels of DR4 and rescued surface DR4 reduction induced by osimertinib (Figure 3F). These data together suggest that osimertinib and other EGFR-TKIs primarily enhance proteasomal degradation of DR4.

### Osimertinib enhances MARCH8-mediated DR4 degradation

**Figure 4:**
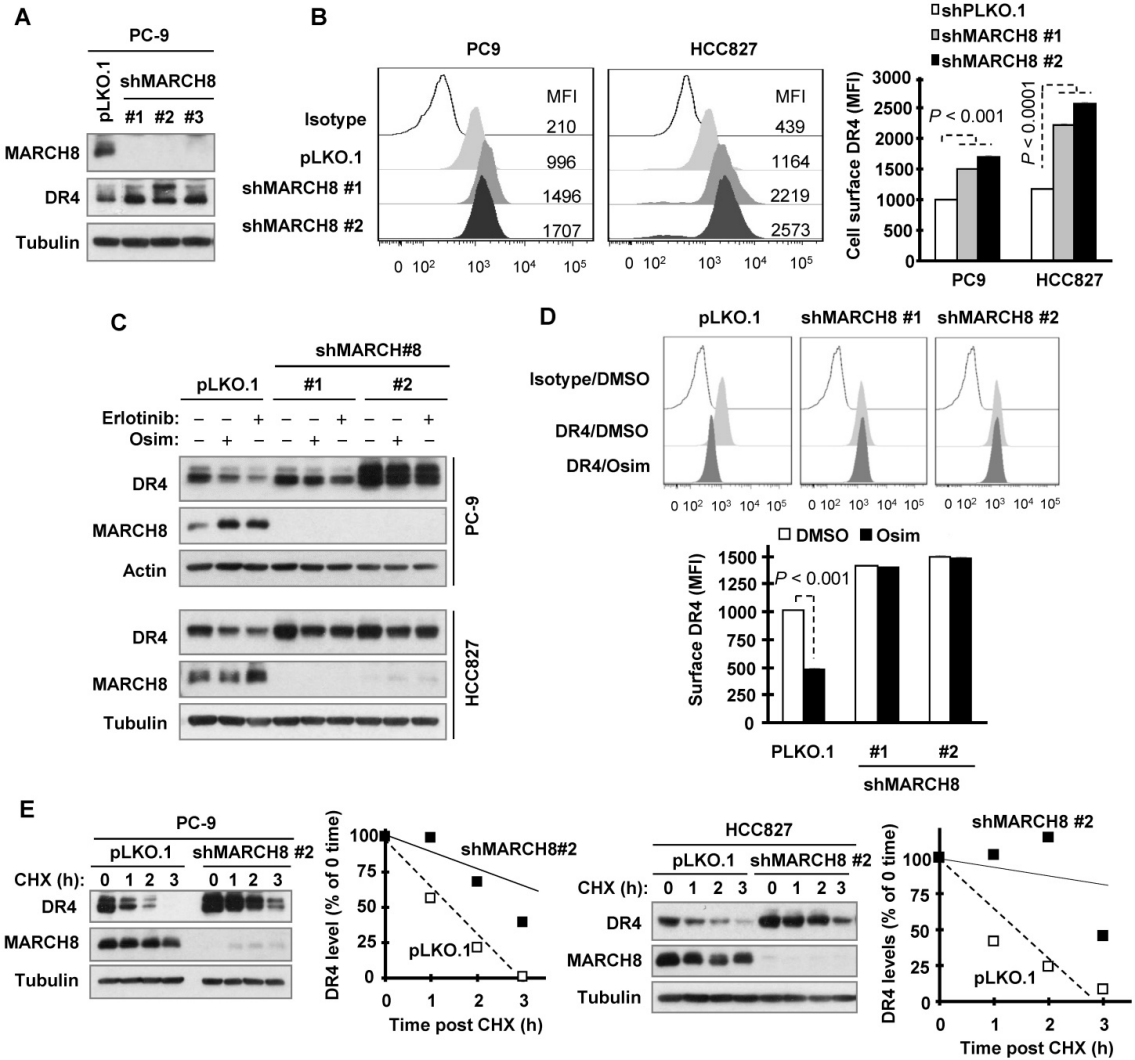
** Knockdown of MARCH8 elevates basal levels of DR4 levels and rescues DR4 reduction induced by osimertinib through preventing its degradation.** *A* and* B*, The indicated cell lines were infected with pLKO.1 or MARCH8 shRNA lentiviruses for 48 h. Western blotting and flow cytometry were used to detect total cellular proteins (*A*) and cell surface DR4 (*B*), respectively. *C*, PC-9 and HCC827 cells expressing shMARCH8 were treated with 100 nM osimertinib or erlotinib for 6 h. MARCH8 knockdown and DR4 levels were detected by Western blotting. *D*, Cell surface DR4 levels of PC-9/shMARCH8 cells exposed to 100 nM osimertinib for 6 h were detected by flow cytometry. The data are means ± SDs of duplicate determinations. *E*, The indicated cell lines were treated with DMSO or 100 nM osimertinib for 6 h followed by addition of 10 µg/ml CHX. Cells were then harvested at the indicated times post CHX for Western blotting. DR4 levels were plotted relative to those at time 0 of CHX treatment (right panels) after being quantified by NIH Image J software and normalized to tubulin.

Since the E3 ligase MARCH8 was suggested to mediate DR4 ubiquitination and degradation and attenuate its cell surface expression 30, we then determined the involvement of MARCH8 in DR4 degradation induced by osimertinib and other EGFR-TKIs. Three different MARCH8 short hairpin RNAs (shRNAs) effectively silenced DR4 expression accompanied with elevated levels of DR4 (Figure 4A). Moreover, knockdown of MARCH8 with two of the shRNAs (#1 and #2) significantly increased cell surface DR4 expression (Figure 4B). Hence MARCH8 indeed modulates basal levels of DR4 levels. Treatment with osimertinib or erlotinib led to reduced DR4 expression in pLKO.l control cells, but not in cell lines infected with either MARCH8 shRNA #1 or #2 (Figure 4C). In agreement, osimertinib significantly decreased cell surface DR4 levels in pLKO.1 control cells, but not in MARCH8 shRNA-expressing cells (Figure 4D). In both PC-9 and HCC827 cell lines, the DR4 degradation rate was much slower in MARCH8 knockdown cells than in pLKO.1 control cells upon treatment with osimertinib (Figure 4E), indicating that MARCH8 knockdown prevents DR4 from degradation induced by osimertinib. These results collectively demonstrate that EGFR-TKIs induce MARCH8-mediated DR4 degradation and attenuation of cell surface DR4 expression.

### DR4 levels are elevated in some osimertinib-resistant cell lines and human lung cancer specimens relapsed from EGFR-TKI treatment

**Figure 5:**
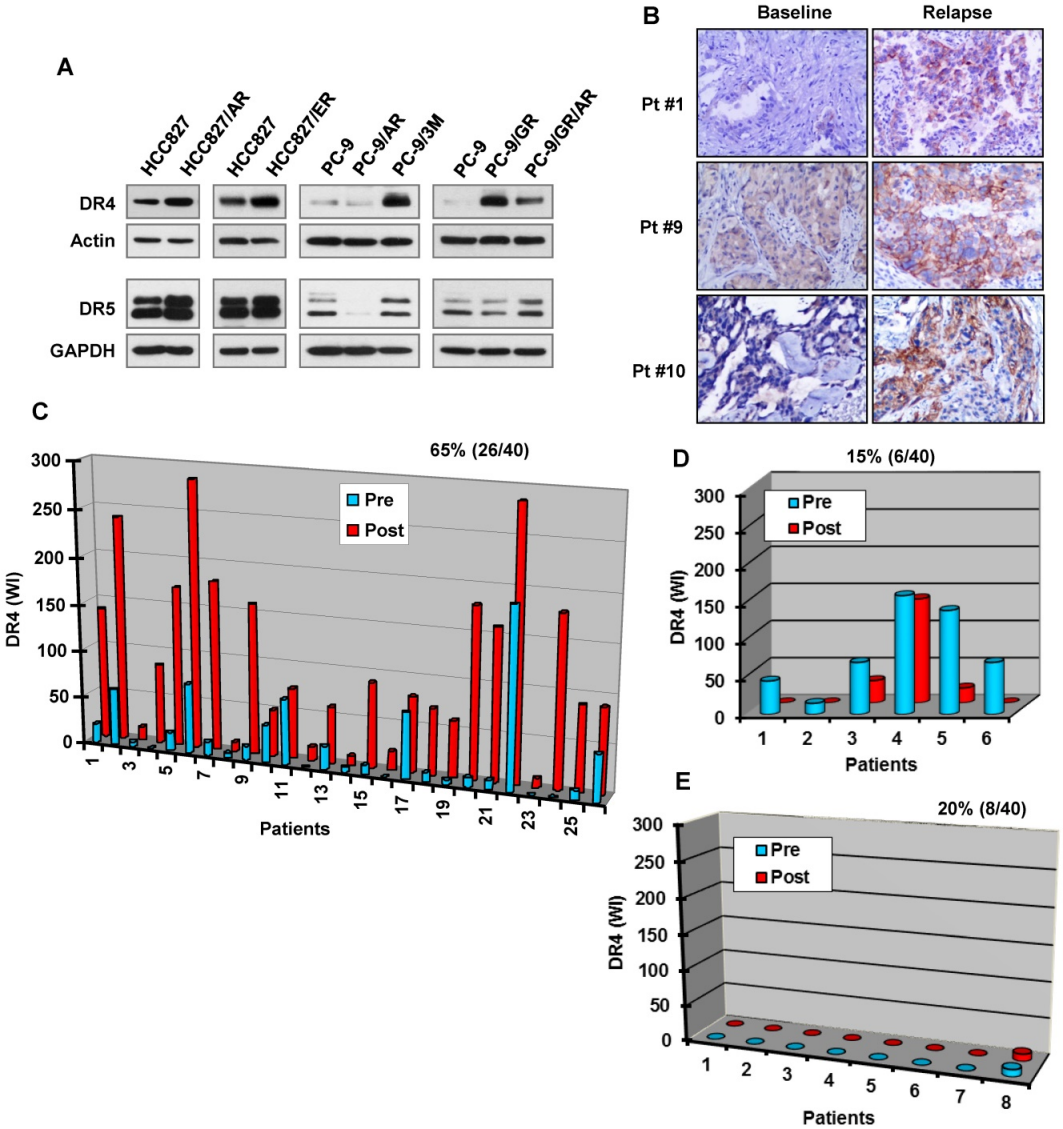
** EGFR-TKI-resistant cell lines and tissues possess elevated DR4 expression.** *A*, The basal levels of DR4 in the indicated cell lines were detected with Western blotting. *B*-*E*, DR4 in human NSCLC tissues with EGFR mutations was detected with IHC. Representative cases in which DR4 expression was increased post relapse from EGFR-TKI treatment are presented in *B*. Quantitation of DR4 expression for all relapsed cases is presented in *C*-*E*. Pt, patient.

Although osimertinib treatment potently decreases DR4 levels in the sensitive EGFRm NSCLC cell lines, we detected much higher levels of DR4 in HCC827/AR, HCC827/ER, PC-9/GR, PC-9/GR/AR and PC-9/3M resistant cell lines than in their corresponding parental cell lines (Figure 5A). In contrast, the levels of DR5 were not or only minimally increased in these resistant cell lines. We further compared DR4 alteration between pre-treatment tumor specimens and tumor tissues collected post relapse to treatment with gefitinib, erlotinib or icotinib. Among 40 paired cases of tissue samples, we detected elevated DR4 expression in 26 cases of post-treatment tissues (65%) and reduced DR4 expression in 6 cases (15%). DR4 expression remained unchanged in 8 cases (20%), among which 7 cases had undetectable DR4 levels (Figures 5B-5E). Hence it is clear that DR4 expression is increased in more than 60% of cases relapsed from EGFR-TKI treatment.

### Modulation of DR4 expression levels alters cell responses to osimertinib-induced apoptosis

**Figure 6:**
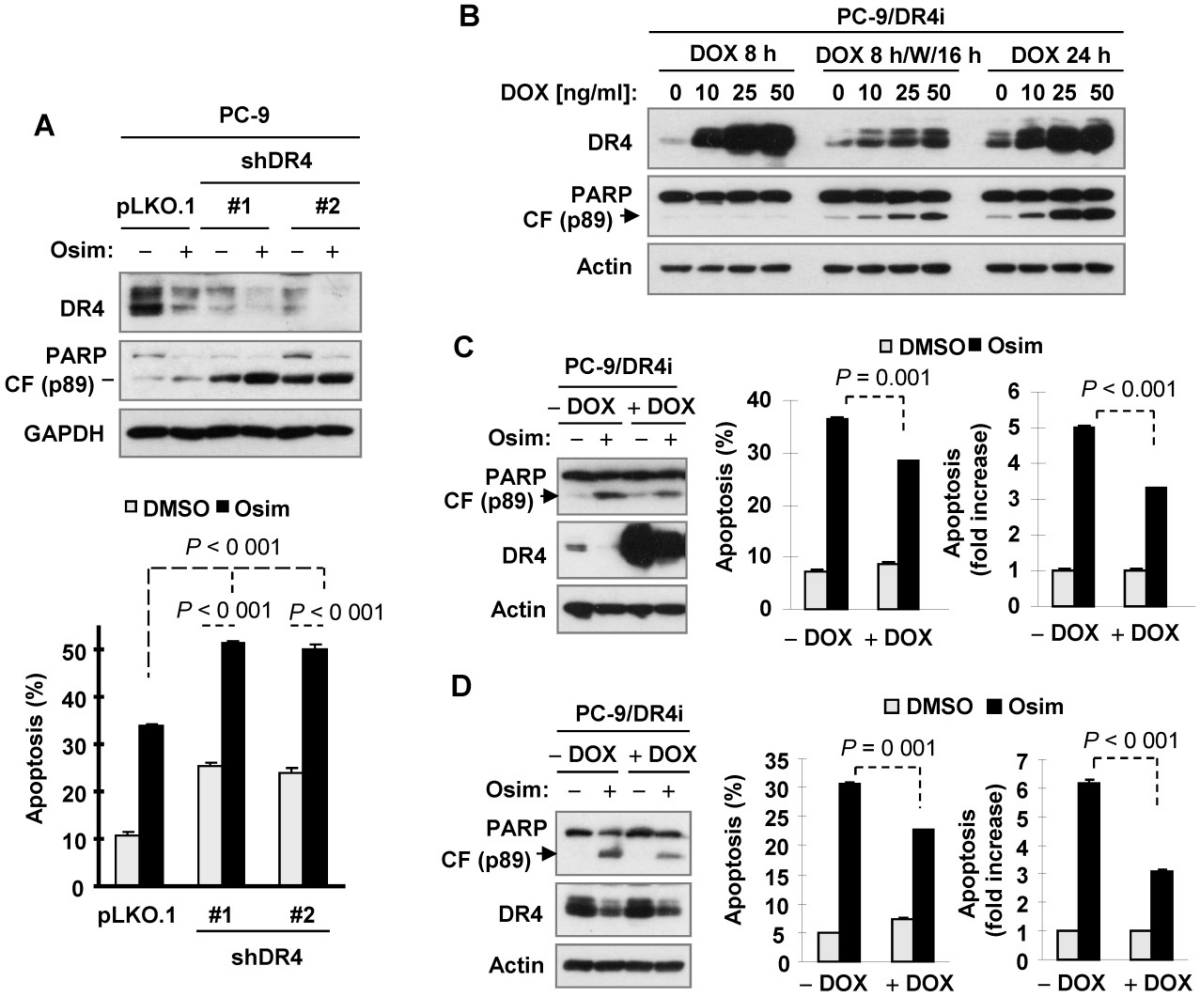
** DR4 knockdown enhances osimertinib-induced apoptosis, whereas induction of ectopic DR4 overexpression transiently compromises osimertinib-induced apoptosis in the sensitive EGFRm NSCLC cell cells.** *A*, Cells were infected with DR4 shRNA lentiviruses for 24 h followed by treatment with 100 nM osimertinib (Osim) for another 24 h. *B*, PC-9/DR4i cells were exposed to the indicated concentration of DOX for 8 h (DOX 8 h), followed by washing and culture with fresh medium for another 16 h (DOC 8 h/W/16 h) or continuously for 24 h (DOX 24 h). *C*, PC-9/DR4i cells were treated with 100 nM osimertinib for 48 h, followed by 20 ng/ml DOX for another 8 h. *D*, PC-9/DR4i cells were treated with 10 ng/ml DOX. After 8 h, the cells were washed with fresh medium and exposed to 100 nM osimertinib for another 24 h. DR4 expression and PARP cleavage were detected by Western blotting and apoptosis was measured by annexin V/flow cytometry. The data are means ± SDs of duplicate determinations (*A*, *C* and* D*). CF, cleaved form.

Given the above observations that DR4 reduction induced by osimertinib or other EGFR-TKIs is accompanied with increased PARP cleavage and tumor growth as demonstrated *in vitro* and *in vivo*, we used DR4 shRNAs to transiently knock down DR4 expression in PC-9 cells followed by treatment with osimertinib and then detected apoptosis. We found that knockdown of DR4 itself significantly increased apoptosis, evidenced by increased PARP cleavage and annexin V-positive cells. When these cell lines were exposed to osimertinib, apoptosis was significantly enhanced in DR4-knockdown cells in comparison with pLKO.1 control cells (Figure 6A). Hence, it is clear that DR4 knockdown induces apoptosis and augments osimertinib-induced apoptosis in PC-9 cells. Moreover, we determined the impact of enforced ectopic DR4 overexpression on osimertinib-induced apoptosis. To avoid the potent cell-killing effect caused by DR4 overexpression that prevents us from making DR4 overexpressing stable cell lines, we established a DR4-inducible cell line from PC-9 cells, named PC-9/DR4i, in which DR4 expression can be controlled by the addition of doxycycline (DOX). As reported previously 37, we found that induction of DR4 overexpression upon DOX treatment for 24 h triggered apoptosis in PC-9/DR4i cells, as evidenced by PARP cleavage. We detected no PARP cleavage in the cells exposed to DOX for a short time (e.g., 8 h) and weak PARP cleavage in cells exposed to DOX for 8 h followed by washing and continuing culture with fresh medium for an additional 16 h (Figure 6B). We thus conducted the following two experiments to demonstrate the impact of enforced DR4 overexpression on osimertinib-induced apoptosis: 1) PC-9/DR4i cells were exposed to DOX for 8 h, washed with medium and then exposed to osimertinib for 24 h; and 2) PC-9/DR4i cells were treated with osimertinib for 40 h followed by co-treatment with DOX for an additional 8 h. Under both conditions, we detected lower amounts of cleaved PARP and fewer annexin V-positive cells in the DOX-exposed cells than in the control cells upon osimertinib treatment (Figures 6C and 6D). Thus, enforced DR4 expression does in part protect the cells from osimertinib-induced apoptosis. Together, these results indicate that modulation of DR4 expression levels affects cell responses to osimertinib-induced apoptosis.

### DR4 knockdown in osimertinib-resistant cells enhances induction of apoptosis by osimertinib, whereas MEK inhibition restores the ability of osimertinib to decrease DR4 levels and augments induction of apoptosis

**Figure 7:**
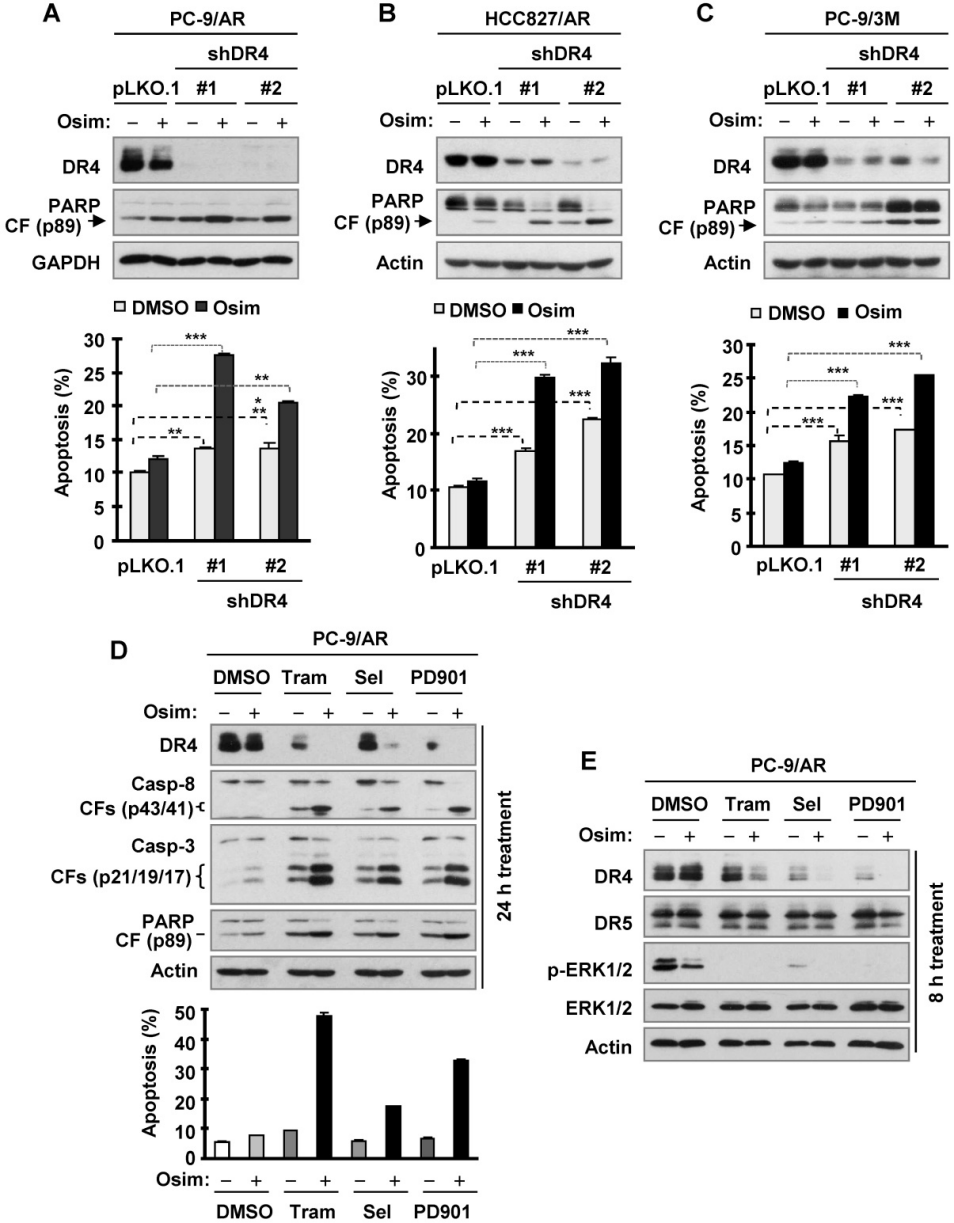
** DR4 knockdown in osimertinib-resistant cell lines sensitizes the cells to osimertinib-induced apoptosis, whereas MEK inhibition combined with osimertinib potentiates DR4 reduction with augmented induction of apoptosis in osimertinib-resistant cells.** *A-C*, The indicated osimertinib-resistant cell lines were infected with DR4 shRNA lentiviruses for 24 h followed by treatment with 100 nM osimertinib (Osim) for another 48 h. DR4 knockdown and PARP cleavage were detected by Western blotting and apoptosis was measured by annexin V/flow cytometry. *D* and *E*, PC-9/AR cells were treated with 100 nM osimertinib alone, 25 nM trametinib (Tram) or 50 nM selumetinib (Sel) or PD0325901 (PD901) alone, and the combination of osimertinib with a given MEK inhibitor for 24 h (*D*) or 8 h (*E*). The cells were then harvested for Western blotting to detect the given proteins (*D* and *E*) and for annexin V/flow cytometric analysis to detect apoptosis (*D*). **, *P* < 0.01; *** *P* < 0.001.CF, cleaved form.

Since osimertinib fails to downregulate DR4 levels in osimertinib-resistant cell lines as described above, we next asked whether enforced downregulation of DR4 sensitizes the resistant cell lines to osimertinib. To this end, we used DR4 shRNAs to transiently silence DR4 expression and then examined its impact on the responses of these cell lines to osimertinib. We conducted these experiments in PC-9/AR, PC-9/3M and HCC827/AR cells and consistently demonstrated that knockdown of DR4 significantly induced apoptosis and enhanced osimertinib-induced apoptosis, as evidenced by increased PARP cleavage and annexin V-positive cells (Figures 7A-C).

We have recently shown that inhibition of MEK with a MEK inhibitor (e.g., trametinib) together with osimertinib restores induction of apoptosis and overcomes osimertinib resistance 35. As reported, the combination of osimertinib with any of the MEK inhibitors, trametinib, selumetinib or PD0325901, enhanced apoptosis as detected by augmented cleavage of caspase-8, caspase-3 and PARP and the appearance of annexin V-positive cells in PC-9/AR cells (Figure 7D). Beyond this expected outcome, all of the tested combinations drastically decreased DR4 levels while treatment with single agent osimertinib or each MEK inhibitor led to little or no reduction in DR4 levels in this cell line at both 8 h and 24 h treatment times (Figures 7D and 7E), clearly indicating an enhanced effect of the combination on downregulation of DR4. Under the same conditions, DR5 levels were not apparently reduced (Figure 7E). Thus, it is clear that the combination of osimertinib with MEK inhibition restores the induction of apoptosis accompanied with an early downregulation of DR4 in osimertinib-resistant NSCLC cells.

### DR4 expression is significantly associated with poor survival of NSCLC patients

**Figure 8:**
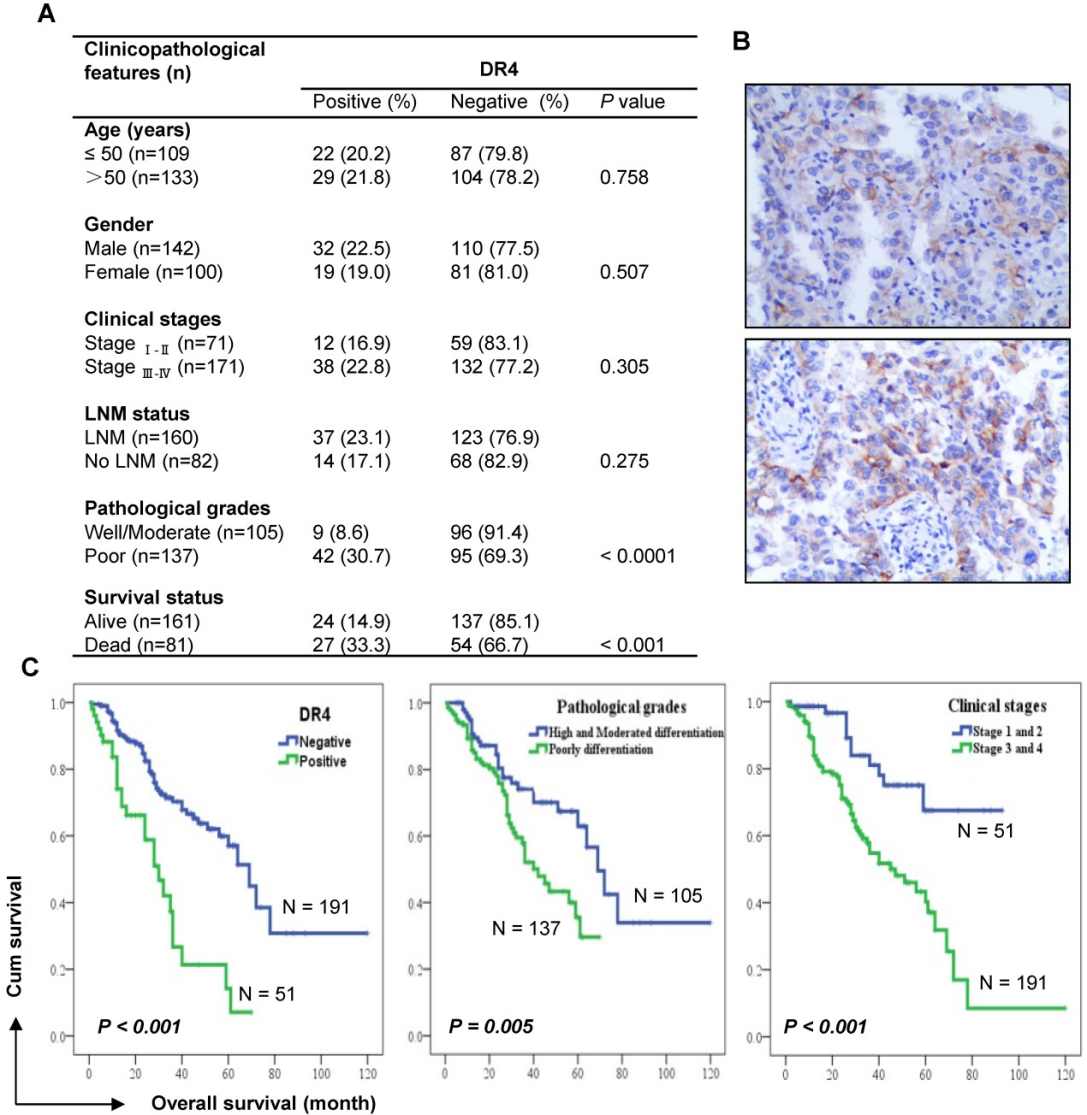
** DR4 expression, like pathological grade and clinical stage, is significantly associated with poor survival of lung adenocarcinoma patients.** *A*, Analysis of the association between DR4 expression and clinico-pathological features of lung adenocarcinomas (n = 242). Chi-square test was used to calculate statistical significance. LNM, lymph node metastasis. *B*, Representative images of DR4 staining. *C*, Kaplan-Meier survival analyses of the impact of DR4 expression, pathological grade and clinical stage on the survival of lung adenocarcinoma patients.

To understand the involvement of DR4 in human NSCLC, we detected DR4 expression using IHC in 242 cases of adenocarcinomas (see supplementary Table S1 for detailed patient characteristics) and examined correlations with patient survival. DR4 expression was significantly higher in poorly differentiated tumors than in well or moderately differentiated tissues. Accordingly, DR4 expression was also significantly higher in tissues from deceased patients than in those from alive patients (Figure 8A). We noted that DR4 positive staining predominantly occurred in the cell membrane (Figure 8B). Kaplan-Meier survival analysis showed that there was a significant inverse correlation between DR4 expression and the overall survival rates of patients (*P* < 0.001): i.e., patients with tumors positive for DR4 expression had significantly shorter overall survival than those with tumors negative for DR4 staining (Figure 8C). The overall survival rates for these lung adenocarcinoma patients were also significantly correlated with conventional prognostic parameters including pathological differentiation grades and clinical stages (Figure 8C). Multivariate analysis considering age, gender, treatment, clinical stage and pathological stage, also showed that DR4 expression, like clinical stage and pathological stage, was significantly associated with the poor survival of patients (Table S2), suggesting that DR4 is an independent prognostic marker, at least for adenocarcinoma patients.

We also determined whether there is a connection between EGFR mutations and DR4 expression in NSCLCs. By analyzing 142 cases of adenocarcinoma with known EGFR mutation status, we found no significant correlation for DR4 expression between EGFR WT and mutant tumors (Table S3), indicating that EGFR mutation does not impact DR4 expression in NSCLCs.

## Discussion

The modulation and role of DR4 in EGFR-targeted cancer therapy has not previously been reported. This study provides the first data revealing a previously undiscovered connection between DR4 modulation and cell response to EGFR-targeted cancer therapy, particularly osimertinib, against EGFRm NSCLCs. Osimertinib and erlotinib reduced DR4 levels accompanied with the induction of apoptosis (e.g., PARP cleavage) in the sensitive EGFRm NSCLC cell lines, but not in insensitive NSCLC cell lines with wild-type EGFR or resistant EGFRm NSCLC cell lines. In agreement with our *in vitro* observations, studies using EGFRm PC-9 xenografts in nude mice showed that DR4 reduction induced by osimertinib occurred far ahead of the apparent induction of apoptosis and inhibition of tumor growth. Together, these data clearly support the tight association between DR4 reduction and cell response to osimertinib or EGFR-targeted cancer therapy against EGFRm NSCLC cells.

Consistent with this association, osimertinib lost its ability to reduce DR4 levels in several osimertinib-resistant EGFRm NSCLC cell lines with distinct resistance mechanisms, including HCC827/AR (*MET* gene amplification and protein hyperactivation), PC-9/3M (C797S mutation) and PC-9/AR (unknown mechanisms). This was also the case for erlotinib. Furthermore, the basal levels of DR4 in most of these resistant cell lines were elevated in comparison with their corresponding parental cell lines. We also detected elevated DR4 expression in > 60% cases of NSCLC tumor tissues relapsed to treatment with first generation EGFR-TKIs including gefitinib, erlotinib and icotinib. Therefore, late rebound DR4 upregulation and loss of response to treatment may represent an important sign of developing resistance. While the elevation of DR4 was detected in most tumor biopsy samples (65%; 26/40) from EGFRm NSCLC patients relapsed to treatment with first generation EGFR-TKIs, DR4 was reduced in 15% of cases (6/40) and remained unchanged in 20 of cases (8/40), among which 7 showed undetectable DR4. Since there are multiple different resistance mechanisms, other mechanisms beyond DR4 may play dominant roles in mediating the acquired resistance in the relapsed tumors where DR4 was undetectable or remained downregulated. Hence, further validation studies with a larger sample size are warranted.

DR4 has long been recognized to be a pro-apoptotic death receptor 10, 11. In this study, transient knockdown of DR4 gene expression caused apoptosis and further enhanced osimertinib-induced apoptosis in the sensitive EGFRm NSCLC cells, whereas enforced DR4 expression in these cells significantly attenuated osimertinib-induced apoptosis. In agreement, transient DR4 knockdown in different osimertinib-resistant cell lines also triggered apoptosis and significantly sensitized these resistant cell lines to osimertinib-induced apoptosis. These findings collectively suggest a previously unrecognized anti-apoptotic function of DR4. We have recently demonstrated that co-inhibition of MEK effectively overcomes acquired resistance to osimertinib via enhancing induction of apoptosis 35. In the current study, the combination of osimertinib with a MEK inhibitor augmented reduction of DR4 in osimertinib-resistant cells accompanied with enhanced induction of apoptosis. This finding further supports the anti-apoptotic role of DR4. Although we currently do not know the underlying mechanisms accounting for the anti-apoptotic function of DR4, further investigation in this aspect is warranted.

It is known that Bim elevation or induction accounts for a critical mechanism by which EGFR-TKIs including erlotinib and osimertinib induce apoptosis in NSCLC cells with EGFR activating mutations 35, 38-40. Osimertinib clearly activates caspase-8, which, in general as DR4 does, works upstream of Bim in EGFRm NSCLC cells and triggers the extrinsic apoptotic pathway as we previously demonstrated 35, 41. Whether there is a connection between DR4 downregulation and Bim-mediated induction of apoptosis in EGFRm NSCLC cells needs further investigation.

DR4 is known to be a TRAIL receptor that can transduce apoptotic signaling 10. Although osimertinib potently decreased DR4 levels including cell surface DR4 levels in sensitive EGFRm NSCLC cells, it still enhanced TRAIL-induced apoptosis in these cell lines as we recently reported 41. Consistently, transient knockdown of DR4 further enhanced TRAIL-induced apoptosis (Fig. S4). In line with this finding, we previously reported that knockdown of DR4 enhanced apoptosis induced by TRAIL or the combination of TRAIL and GGTI-298 (a geranylgeranyltransferase I inhibitor) in NSCLC cells 17. Interestingly, these cell lines became less sensitive to TRAIL once becoming resistant to osimertinib and lost response to the combination of osimertinib and TRAIL in comparison with their corresponding parental cell lines 41, despite the elevated basal levels of DR4 as demonstrated in this study. TRAIL-induced activation of the extrinsic apoptotic pathway in TRAIL sensitive cells plays an important role in the immune surveillance of tumors and metastases 12, 14, 42-46. However, endogenous TRAIL/death receptor interaction in TRAIL-resistant cancer cells may activate NF-κB signaling and induces inflammatory cytokine (e.g., CCL2) secretion, which inactivates immune cells and promotes a tumor-supportive immune microenvironment and tumor growth as recently demonstrated 32, 33. Therefore, in addition to the direct effects of osimertinib on EGFRm NSCLC cells including induction of apoptosis, an indirect effect on enhancing the immune clearance of EGFRm NSCLC cells may also be an important mechanism accounting for osimertinib's therapeutic efficacy. Accordingly, resistance to this immune clearance may contribute to the development of acquired resistance. Hence, further investigation in this direction is under consideration.

In this study, we have demonstrated that EGFR-TKIs such as osimertinib decrease DR4 levels through both transcriptional and posttranslational mechanisms. The transcriptional regulation involves suppression of MEK/ERK/AP-1-dependent transcription of DR4, whereas the posttranslational regulation is associated with enhancement of MARCH8-mediated DR4 degradation (see summary schema in Figure S5). Moreover, we noted that both osimertinib and erlotinib increased MARCH8 levels in EGFRm NSCLC cells (Figure 4C). Further detailed studies of MARCH8 upregulation induced by osimertinib and other EGFR-TKIs including the underlying mechanisms are ongoing. In parallel to the suppression of MEK/ERK signaling, osimertinib potently suppressed Akt phosphorylation in both PC-9 and HCC827 cells (Figure S6), which was also documented in our recent study 47. Whether there is a connection between suppression of PI3K/Akt signaling and downregulation of DR4 is unknown and needs further investigation.

It was previously suggested that MARCH8 primarily mediates lysosomal degradation of DR4 since proteasome inhibitors exerted limited protective effects on rescuing MARCH8-induced DR4 degradation 30. In the current study, reduction of DR4 including cell surface DR4 induced by EGFR-TKIs including osimertinib, CO1686 and erlotinib could be effectively rescued by the proteasome inhibitor MG132, but only partially or minimally by the tested lysosome inhibitors chloroquine and Baf A1. Hence, it is apparent that EGFR-TKIs primarily induce MARCH8-mediated proteasomal degradation of DR4 in the sensitive EGFRm NSCLC cells.

Previous studies with clinical cancer tissues have shown that DR4 is highly expressed in breast cancer patients with invasive ductal carcinoma 48 and its high expression in stage III adjuvant-treated colon cancer patients is associated with worse disease-free and overall survival 49. Our current study with human lung adenocarcinoma tissues has also demonstrated that DR4 protein expression is significantly associated with poor survival of patients, suggesting a poor prognostic function of DR4. Therefore, the involvement of DR4 in cancer is likely to be complicated and beyond what we have previously known about its pro-apoptotic function.

In summary, the current study has demonstrated that DR4 expression is a poor prognostic factor in human lung adenocarcinoma and revealed a previously undiscovered anti-apoptotic function of DR4 in the induction of apoptosis by osimertinib and other EGFR-TKIs. Early DR4 reduction is likely to be a predictive sign of response of EGFRm cancer cells or tumors to osimertinib; however, the later stage of rebound upregulation of DR4 suggests resistance to osimertinib treatment. Development of approaches to enforce DR4 suppression may provide an effective strategy to overcome acquired resistance to osimertinib or other EGFR-TKIs although they may be different from previous approaches that develop DR4 agonists, such as DR4 agonistic antibodies aiming to enhance apoptosis through induction of DR4 trimerization. Moreover, the complex biological function of DR4 in cancer needs further investigation.

## Materials and Methods

### Reagents

The resources and preparation of osimertinib, CO1686, erlotinib, selumetinib (AZD6244), PD0325901, trametinib (GSK1120212), MG132, actinomycin D (Act D) and CHX were the same as described previously 35, 50. Bafilomycin A1 (Baf A1) was purchased from LC laboratories (Woburn, MA). Mouse (B-N28) and rabbit (D9S1R) monoclonal DR4 antibodies were purchased from Cell Science (Newburyport, MA) and Cell Signaling Technology, Inc. (Beverly, MA), respectively. Rabbit monoclonal DR5 antibody (D4E9) was purchased from Cell Signaling Technology. Other antibodies were the same as described in our previous studies 29, 35, 50, 51.

### Cell lines and cell culture

HCC827/AR (AZD9291-resistant with *c-MET* amplification), HCC827/ER (erlotinib-resistant with *c-MET* amplification), PC-9/GR (gefitinib-resistant with T790M mutation), PC-9/AR (AZD9291-resistant), PC-9/GR/AR (gefitinib- and AZD9291-resistant), PC-9/3M (19del, T790M and C797S triple mutations) and other cell lines used in this study and culture conditions were the same as described previously 35, 50. These cell lines were not genetically authenticated. Mycoplasma test was performed regularly or as needed using MycoAlert@ Mycoplasma Detection Kit (Lonza; Rockland, ME).

### Detection of apoptosis

Apoptosis was evaluated with an Annexin V/7-AAD apoptosis detection kit (BD Biosciences; San Jose, CA) according to the manufacturer's instructions. Caspase and PARP cleavage were also detected by Western blot analysis as additional indicators of apoptosis.

### Cell surface DR4 detection

Cell surface DR4 expression was detected with flow cytometry as described previously 52.

### Western blot analysis

Preparation of whole-cell protein lysates and Western blot analysis were described previously 35, 50.

### Gene knockdown using shRNA

Lentiviral DR4 (#1 and #2) and MARCH8 (#1 and #2) shRNAs in pLKO.1 were purchased from Open Biosystems (Huntsville, AL) and Sigma (St. Louis, MO), respectively, and used according to the manufacturer's instructions.

### Generation of lentiviral DOX-inducible expression system

Lentiviral vector FUGW vector 53 was used as backbone to generate a Tet-on inducible DR4 construct. rtTA responsive promoter TREmiCMV driving the expression of DR4 transcript was PCR-amplified from Clontech's pTRE-Tight vector and inserted between HIV-Flap and ubiquitin promoter. The reverse transactivator rtTA2S-M2, which was obtained from Dr. A. Chan (Department of Human Genetics, Emory University, Atlanta, GA), was inserted after ubiquitin promoter. In order to generate Tet inducible stable cell lines, IRES/puromycin sequence (PCR-amplified from Clontech's pIRESpuro vector) was inserted immediately after rtTA2SM2 to express the bicistronic transcript driven by an ubiquitin promoter (Figure S7). DR4 induction lentiviruses were then generated by transfecting 293T cells with 3 μg lentiviral vector carrying inducible DR4 gene, 2.7 μg ΔR8.92 and 0.3 μg CMV-VSVG using Polyjet. Lentiviruses were then harvested as described above. The tested cells (e.g., PC-9) were infected by lentiviruses using the infection cocktail. After 48 h, the infected cells were selected using 2 μg/ml of puromycin for 3 days and cultured for further use (e.g., PC-9/DR4i).

### mRNA detection

DR4 mRNA was detected with RT-PCR as described previously 28. Moreover, mRNA alterations were also detected with RNA-seq, which was conducted using NovaSeq sequencer in MedGenome Inc., (Foster City, CA). Differential gene expression analysis was performed using DESeq2.

### Reporter plasmids and luciferase assay

All *DR4* reporter constructs used in this study and the luciferase assay were the same as described previously 23.

### Human NSCLC tissues

The paired tissue samples from EGFR mutant NSCLC patients before treatment (i.e., baseline) and after relapse from treatment with first generation EGFR-TKIs including gefitinib, erlotinib or icotinib were primarily collected at the Second Xiangya Hospital (Changsha, Hunan, China), Henan Cancer hospital (Zhengzhou, Henan, China) and Daping Hospital (Chongqing, China) under the Ethics Review Committee (IRB)-approved protocols (2019-009, 2019-067 and 2019-274, respectively). An additional 242 cases of tumor samples were obtained from lung adenocarcinoma patients who underwent surgical treatment in Department of Thoracic Surgery from 2003 to 2013 through Department of Pathology at the Second Xiangya Hospital of Central South University (Changsha, China). These patients had been submitted to routine staging and definitive surgical resection of the lung and systematic mediastinal lymph node dissection. All patients had a confirmed histological diagnosis of NSCLC according to the WHO histological classification of lung cancer. The staging classification of the current analysis was carried out based on the criteria of the 7th edition of the AJCC/UICC TNM staging system of lung cancer. No patients had been previously treated with chemotherapy and radiotherapy at the time of initial surgery. Complete clinical record and follow-up data were available for all patients. Overall survival time was calculated from the data of diagnosis to the date of death or the data last known alive. The mean follow-up period is 35.9 months (6-120 months). Written informed consent was obtained from these patients, and this study was approved by the IRB of the Xiangya Hospital of Central South University (S-02/2000).

### Immunohistochemistry (IHC)

Human NSCLC tissues were stained with IHC using the EnVision™ + Dual Link System-HRP Kit (Dako; Carpinteria, CA). The rabbit monoclonal antibody against DR4 (D9S1R; #42533) was purchased from Cell Signaling Technology (Danvers, MA 01923) and used at 1:100 dilutions overnight at 4^o^C. The specificity of the antibody was determined with matched IgG isotype antibody as a negative control in IHC. Moreover, a single band of correct molecular weight in Western blotting was assured. Both the percentage of positive staining in tumor cells and intensity of staining were scored. The intensity of IHC staining was measured by using a numerical scale (0 = no expression, 1 = weak expression, 2 = moderate expression and 3 = strong expression). The staining data were finally quantified as the weighted index (WI) (WI = % positive staining in tumor x intensity score) as previously described 54. DR4 staining was scored as negative (≤ 10 WI) and positive staining (> 10 WI), respectively. The WI was determined by 2 individuals, and the final values were the average of the two readings. cPARP in xenograft tissues were stained with cPARP antibody purchased from Cell Signaling Technology (#5625) at 1:50 dilution.

### Animal xenograft and treatments

Animal experiments were approved by the Institutional Animal Care and Use Committee (IACUC) of Emory University and conducted as described previously 50. In short, 5 to 6 week old female athymic (nu/nu) mice purchased from Charles River Labs (Wilmington, MA) received subcutaneous injections of PC-9 cells (2 × 10^6^/mouse) in serum-free medium in the flank region of the nude mice. When tumors reached a size of approximately 300 mm^3^, the mice were randomized into two groups (n = 12/group) according to tumor volumes and body weights for the following treatments: vehicle control and osimertinib (10 mg/kg/day, og). Tumor volumes were measured using caliper measurements and calculated with the formula *V* = π(length × width^2^)/6. On days 1, 3, 6 and 12 post treatment, 3 mice in each group were euthanized with CO_2_ asphyxia. The tumors were then removed, weighed and frozen in liquid nitrogen. Tumor tissue aliquots were homogenized in protein lysis buffer for preparation of whole-cell protein lysates for Western blotting to detect the given proteins.

### Statistical analysis

The statistical significance of differences between two experimental groups was analyzed with two-sided unpaired Student's *t* tests (for equal variances) or with Welch's corrected *t* test (unequal variances) by use of Graphpad InStat 3 software. Results were considered to be statistically significant at *P* < 0.05.

## Supplementary Material

Supplementary figures and tables.

## Abbreviations

DR4: death receptor 4
NSCLC: non-small cell lung cancer
EGFR: epidermal growth factor receptor
EGFR-TKIs: EGFR-tyrosine kinase inhibitors
EGFRm: EGFR mutant
WT: wild-type
TRAIL: tumor necrosis factor-related apoptosis-inducing ligand
MARCH8: membrane-associated RING-CH-8
CHX: cycloheximide
shRNA: short-hairpin RNA
IHC: immunohistochemistry
