# Supplementary material for: Downregulation of death receptor 4 is tightly associated with positive response of EGFR mutant lung cancer to EGFR-targeted therapy and improved prognosis
Source: Theranostics. 2021 Feb 15;11(8):3964–80. doi: 10.7150/thno.54824 (PMC7914351; doi:10.7150/thno.54824)
Supplement: Supplementary file 1 — Supplementary figures and tables. [file thnov11p3964s1.pdf]

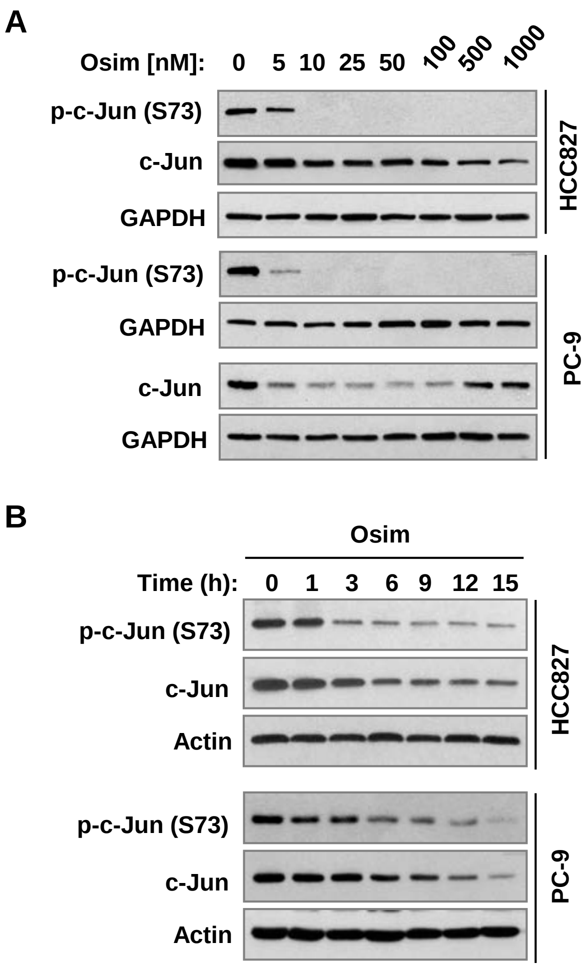

Figure S1. Osimertinib decreases the levels of c-Jun and p-c-Jun in EGFRm NSCLC cells. The indicated cell lines were exposed to different concentrations of osimertinib (Osim) for 12 h (*A*) or to 100 nM osimertinib1 for different times (*B*). The given proteins were detected with Western blotting.

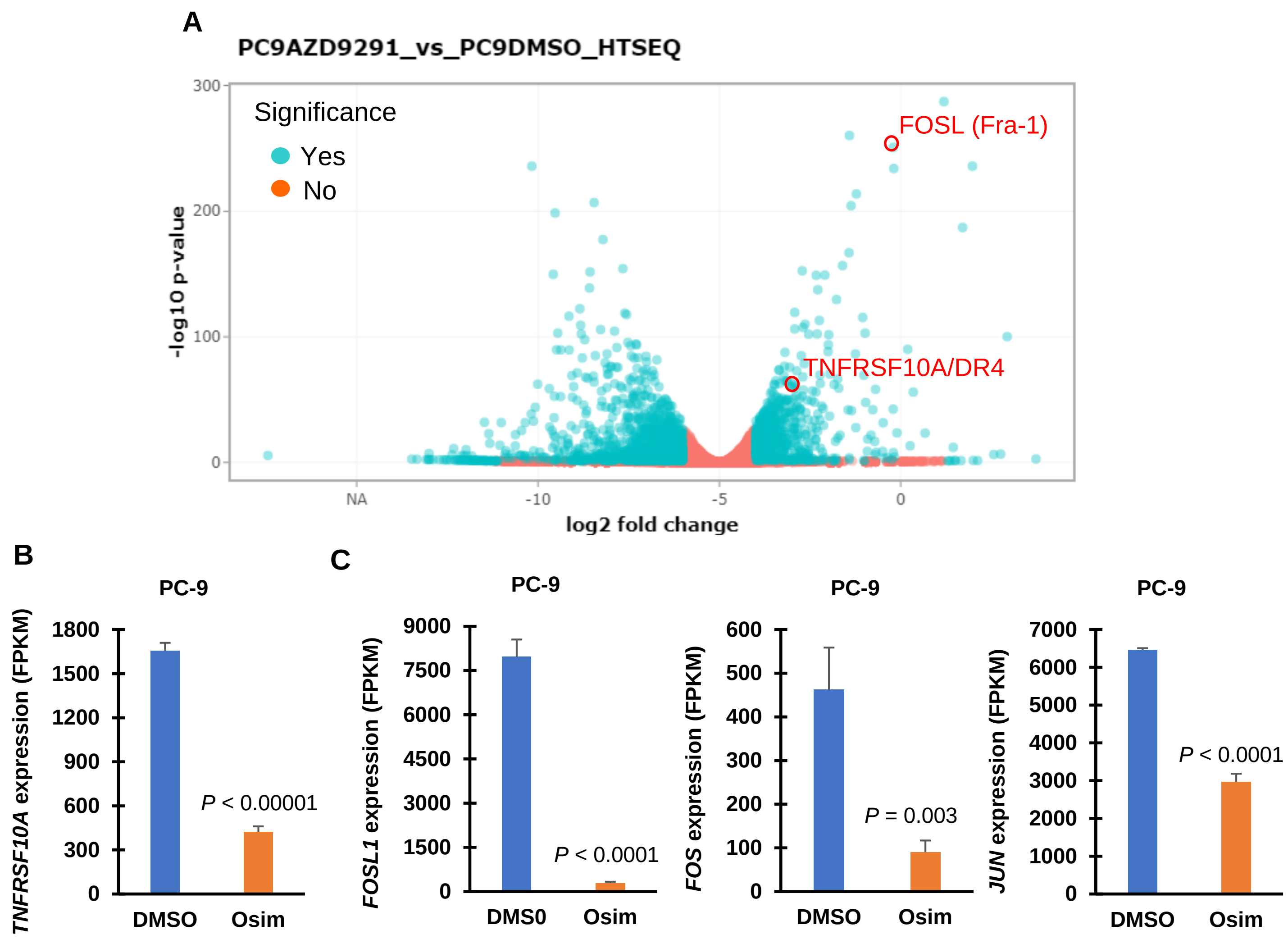

Figure S2. Downregulated expression of *DR4* (*TNFRSF10A*) and other AP-1 associated genes in osimertinib-treated EGFRm NSCLC cells detected in RNA-seq analysis. PC-9 cells were exposed to DMSO or 100 nM osimertinib (Osim) for 14 h and then harvested for RNA-seq analysis. The expression values for each gene were presented in FPKM (fragments per kilobase per million) units.

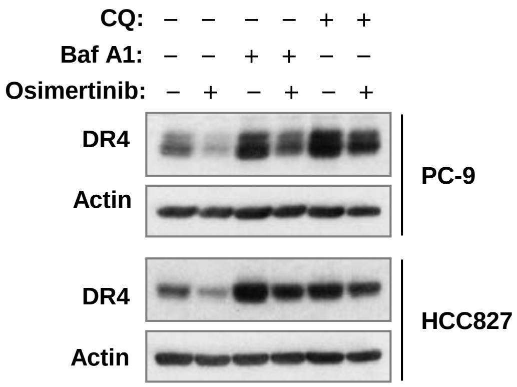

Figure S3. The lysosomal inhibitors partially or minimally rescue DR4 reduction induced by osimertinib in EGFRm NSCLC cells. The indicated cells were pre-treated with 20 µM CQ or 50 nM Baf A1 for 1 h and then co-treated with 100 nM osimertinib for an additional 6 h. Total cellular DR4 and other proteins were detected with Western blotting.

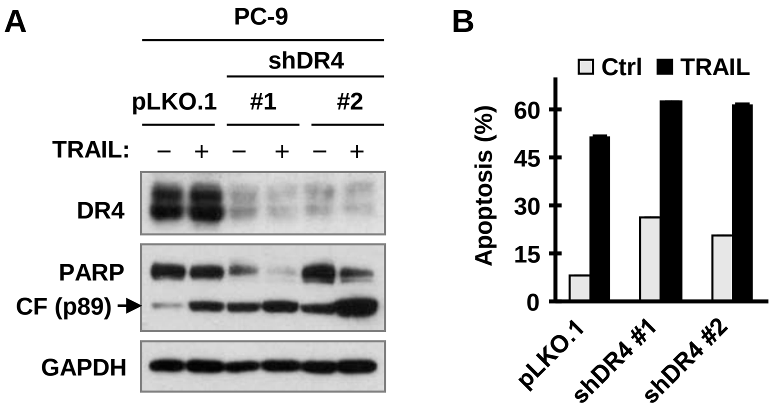

Figure S4. DR4 knockdown enhances TRAIL-induced apoptosis. PC-9 cells were infected with DR4 shRNA lentiviruses for 36 h followed by treatment with 15 ng/ml TRAIL for another 12 h. DR4 expression and PARP cleavage were detected by Western blotting (*A*) and apoptosis was measured by annexin V/flow cytometry (*B*). The data are means ± SDs of duplicate determinations (*B*). CF, cleaved form.

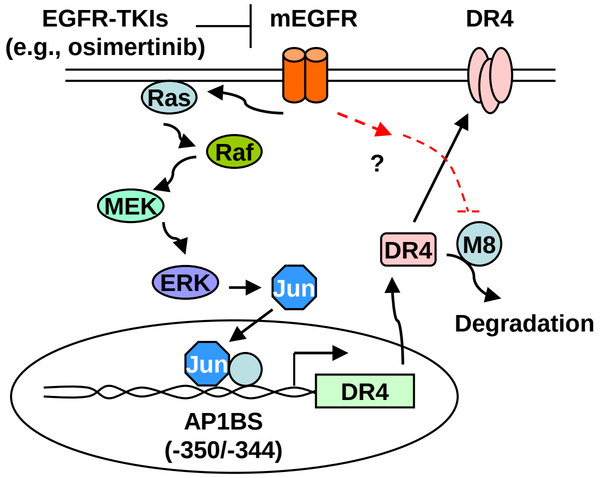

Figure S5. A working model for DR4 suppression by EGFR-TKIs such as osimertinib involving both transcriptional and post-translational mechanisms. M8, MARCH8. BS, binding site.

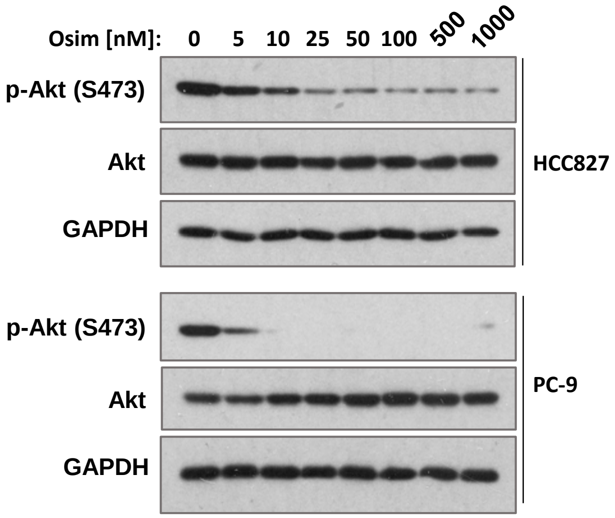

Figure S6. Osimertinib suppresses Akt phosphorylation in EGFRm NSCLC cell lines. Both PC-9 and HCC827 cell lines were exposed to different concentrations of osimertinib (Osim) as indicated for 8 h. The interested proteins were detected with Western blotting.

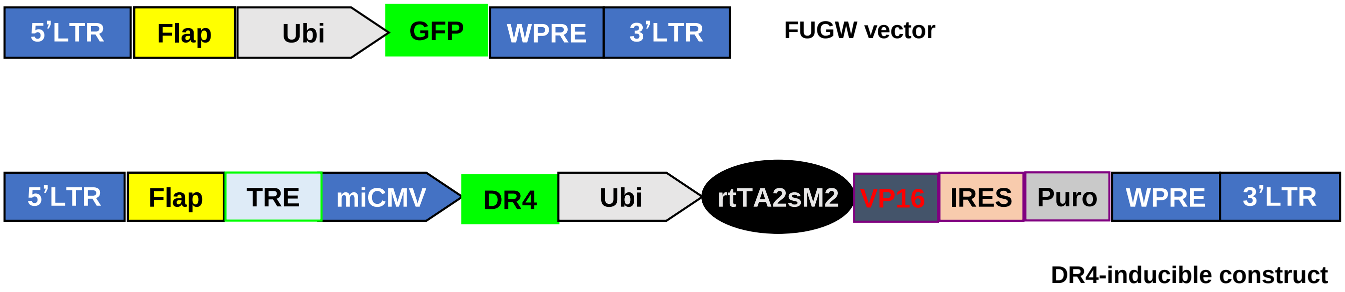

Figure S7. Schema for the construction of lentiviral inducible DR4 expression plasmid based using FUGW vector as backbone. All individual fragments were amplified by PCR and inserted into backbone vector.

Table S1. Clinical features of 242 cases of lung adenocarcinoma

Patients characteristics	No. of patients (%)
**Age (years)**	
≤ 50	109 (45.0)
＞50	133 (55.0)
**Gender**	
Male	142 (58.7)
Female	100 (41.3)
**Clinical stages**	
Stage Ⅰ	9 (3.7)
Stage Ⅱ	62 (25.6)
Stage Ⅲ	131 (54.1)
Stage Ⅳ	40 (16.6)
**Lymph node status**	
N0	82 (33.9)
N1/N2/N3	160 (66.1)
**Pathological grades**	
Well	16 (6.6)
Moderate	89 (36.8)
Poor	137 (56.6)
**Survival status**	
Live	161 (66.5)
Death	81 (33.5)

Table S2. Summary of multivariate analysis of Cox proportional regression for overall survival in 242 cases of lung adenocarcinoma

Parameter	B	SE	Wald	Sig.	Exp (B)	95% CI for Exp (B)	
						Lowe	Upper
Age	-.059	.246	0.57	.811	.943	0582	1.528
Gender	.000	.236	.000	.999	1.000	.630	1.589
Treatments	-.237	.133	3.152	.076	.789	.607	1.025
Clinical stages	.539	.136	15.756	.0001*	1.715	1.314	2.238
Pathological grade	.753	.237	10.096	.001*	2.122	1.334	3.376
DR4 expression	.969	.260	13.859	.0001*	2.635	1.582	4.389

Abbreviations: CI, confidence interval; B, beta coefficient; SE, standard error; Sig., significance; Exp (B), exponentiation of the B coefficient.
*, statistically significant in multivariate analysis of Cox regression.

Table S3. Association between DR4 expression and EGFR mutation status in lung adenocarcinoma (n=124)

			DR4		
			Negative	Positive	*P*-values (Sig)
EGFR	WT	N (%)	35 (68.6)	16 (31.4)	1.876 (0.171)
	Mutant	N (%)	58 (79.5)	15 (20.5)	
